# Reducing False Negative Reads in RFID Data Streams Using an Adaptive Sliding-Window Approach

**DOI:** 10.3390/s120404187

**Published:** 2012-03-28

**Authors:** Libe Valentine Massawe, Johnson D. M. Kinyua, Herman Vermaak

**Affiliations:** 1 School of Electrical and Computer Systems Engineering, Central University of Technology, Free State, Bloemfontein 9301, South Africa; E-Mail: hvermaak@cut.ac.za; 2 School of Computer and Information Systems, Virginia International University, Fairfax, VA 22030, USA; E-Mail: johnson.kinyua@campus.viu.edu

**Keywords:** data filtering, data cleaning, RFID, RFID middleware, sliding-window filter

## Abstract

Unreliability of the data streams generated by RFID readers is among the primary factors which limit the widespread adoption of the RFID technology. RFID data cleaning is, therefore, an essential task in the RFID middleware systems in order to reduce reading errors, and to allow these data streams to be used to make a correct interpretation and analysis of the physical world they are representing. In this paper we propose an adaptive sliding-window based approach called WSTD which is capable of efficiently coping with both environmental variation and tag dynamics. Our experimental results demonstrate the efficacy of the proposed approach.

## 1. Introduction

Radio frequency identification (RFID) is a technology that allows an object, a place or a person to be automatically identified with neither physical nor visual contact. Although RFID technology is not new [1], the current upsurge interest in this technology stems from the development of low-cost passive RFID tags and vigorous RFID standardisation efforts. RFID has recently been deployed in many different fields which include: distribution logistics, pharmaceutical and health care, library, contactless ID cards and tickets, asset management, manufacturing, the garment and automotive industries, animal identification, traffic applications, the aviation industry, the military and many more [2,3].

Although the performance of UHF passive RFID-based systems was improved significantly by the introduction of the EPC Class-1 Generation-2 protocol (C1G2) [4], several studies on the performance of the C1G2 RFID systems indicates that the overall performance of the system is still implementation dependent [5–8]. The empirical study of UHF RFID performance by Buettner *et al.* [6] shows that physical effects such as errors and multipath interference are significant factors that degrade the overall performance of commercial readers. These effects increase both the duration of each reader cycle and the number of cycles to read all tags in a tag set. They argue that the error rates are highly location dependent and the level of degradation is implementation specific. The work by Kawakita *et al.* [8] shows that the bit errors, due to erroneous communication links, significantly degrade C1G2 performance. In actual UHF passive RFID deployment, the RFIDs usually share the frequency band with other UHF wireless devices as well as neighbour RFIDs. While some interferences are predictable and controllable, others are unpredictable and uncontrollable, such as that due to mobile wireless devices. Therefore, despite the improvements on tag detection rates by using the C1G2 protocol, factors such as tag-reader configurations, multipath and unpredictable interferences in the deployment environment still contribute to degradation of the performance and reliability of the RFID system leading to noisy and incomplete data. RFID data cleaning is, therefore, essential in order to correct the reading errors, and to allow these data streams to be used to make correct interpretations and analysis of the physical world they are representing.

Methodologies for improving reliability of RFID data proposed in the literature can be divided into three main categories: physical solutions, middleware solutions and deferred solutions [9]. Physical solutions include improvement of hardware performance to improve the reliability of the data such as those described in [10]. Redundant techniques include using multiple tags and readers to identify the same object [11,12], and additional techniques to remove duplicate readings generated from such deployments [13–15]. Middleware solutions include algorithms to correct the incoming sensor data streams before the data is passes into the database [16–18]. The deferred solutions incorporate intelligent techniques which correct the data in the later stages within the data storage [9,19]. Our work falls in the category of middleware-based solutions; specifically window-based smoothing methods. We decided to use the window-based method because of their simplicity and our work extends the work proposed by Jeffrey *et al.* [16].

Many commercial RFID-middleware solutions [20,21] contain a fixed temporal-based sliding-window data-smoothing filter as a solution to RFID unreliability, and applications are required to set the window size. The goal is to reduce or eliminate dropped readings by giving each tag more opportunity to be read within the smoothing window. The study by Jeffery *et al.* in [16] shows that setting the appropriate smoothing-window size is a non-trivial task, especially in the mobile environment. It requires a carefully balance between the two opposing application requirements, which are: (1) to *ensure completeness* for the set of tag readings due to tag-reader system unreliability; and (2) to *capture tag dynamic* due to tag movement in and out of the reader's detection region. Large window sizes are good in ensuring completeness by smoothing out the missed readings, but they are not efficient in detecting tag transitions. On the other hand small window sizes are able to detect transitions, but they are not capable of compensating for the missed readings. Small windows lead to *false negative* errors in which the tag is mistakenly assumed to be absent while it is actually present. In the mobile tag environment big window sizes, while trying to compensate for the missed readings, introduce other errors which are known as *false positive* errors, which are readings in which the tags are mistakenly assumed to be present while they have already exited the detection region. False positive readings in our discussion are, therefore, by-products of the cleaning scheme caused by interpolation of readings within the big window size. Experimental results in [16] show that there is no single window size that can perform consistently well under variable environmental conditions. Taking into consideration the sensitivity of the tag-reader performance on the deployment environment, this means that a small change in the environment can render the initially optimised cleaning window unable to smooth the data.

Jeffery *et al.* [16] proposed an adaptive sliding-window cleaning method called Statistical sMoothing for Unreliable RFid data (SMURF). SMURF models the unreliability of RFID readings by viewing RFID streams as a statistical sample of tags in the physical world, and exploits techniques grounded in sampling theory to drive its cleaning processes. SMURF does not expose the smoothing-window parameter to the application; instead it automatically determines the most appropriate window size and continuously adapts it over the lifetime of the system based on observed readings. Adopting the statistical approaches proposed in SMURF, we developed our own adaptive cleaning scheme for RFID data streams, called WSTD, with a more efficient transition detection mechanism. WSTD is able to adapt its window size to cope with fluctuations of the tag-reader performance due to changes in the environment, while relatively accurately detecting the transition points. This is an integral part of our ongoing work on developing multi-agent based RFID middleware systems [22,23]. WSTD is used as a data cleaning mechanism for low-level RFID data processing tasks within our middleware system.

The remainder of this paper is structured as follows: Section 2 describes the statistical sampling perspective of RFID data streams. Section 3 describes the proposed WSTD algorithm to efficiently detect transition. Section 4 evaluates the performance of WSTD in comparison with SMURF; followed by conclusions in Section 5.

## 2. Statistical Sampling Perceptive of RFID Data Streams

According to the RFID reader-tag performance analysis presented in [5–7], the raw RFID data streams do not provide a correct representation of the physical world that they are representing. A significant number of tags which are within the reader's read range are not consistently read by the reader due to either their orientation with respect to the reader, distance from the reader, presence of metal, dielectric or water material close to the tag and other factors. These missing tags imply that typically only a *subset* of the tag population is actually observed on every read cycle. Therefore, the observed RFID readings can be viewed as a random sample of the population of tags in the physical world. The key insight is viewing each read cycle output as a random sampling trial and the smoothing window as repeated random sampling trials [16]. In our discussion we will refer to an atomic unit of time used by one read cycle as an *epoch*.

Let *Nt* denote the unknown size of the underlying tag population at epoch *t* and let *S_t_* ⊆ {1, …, *N_t_*} denote the subset of the tags observed (“sampled”) during that epoch. *S_t_* can be viewed as unequal probability random sample of the tag population. Probability *p_i,t_* of selecting *tag i* at epoch *t* can be calculated from the epoch *_t_* output information using the number of reads (tag responses) for tag *i* in combination with the known number of interrogation cycles (number of requests) using Equation (1):

(1) $$p_{i,t}=\frac{\text{number of responses}}{\text{number of requests}}$$

## 3. Window Sub-Range Transition Detection (WSTD) Scheme

In this section, we present our WSTD cleaning scheme. WSTD uses binomial sampling concepts to calculate the appropriate window size and π-estimator to estimate the number of tags as proposed by SMURF. WSTD then uses the comparison of the two window sub-range observations or estimated tag counts and some rules to detect when transition occurs within the window and then adjust the window size appropriately.

Our algorithms make the following assumptions: firstly, in our data model the probability of detection of the tag will be the same, given that the tag-distance and the environment conditions are the same. However, in real world RFID data does not exactly follow this model. Occasionally, a tag placed at a specific distance relative to the reader in a specific environment will cause the reader to generate readings with varying probability of detection. Secondly, since our transition detection mechanism is based on the characteristics of the underlying data stream, such variations in detection rate, may lead to false transition detection. Nevertheless, such behavior occurs rarely, and with improvements of tag-reader performance we expect it to have a minimal negative effect in practice. We first present how WSTD cleans individual tag data and then present how it cleans tag aggregates in the applications which only need to know the number of tags available.

### 3.1. Adaptive Individual Tag Cleaning

#### 3.1.1. Completeness Requirement

Each epoch is viewed as an independent Bernoulli trial (*i.e.*, a sample draw for *tag i*) with success probability *p_i,t_* using Equation (1) [16]. This implies that the number of successful observations of *tag i* in the window *W_i_* with *w_i_* epochs (*i.e., W_i_=* (*t* − *w_i_, t*)) is a random variable with a binomial distribution *B*(*w_i_, p_i,t_*). In the general case, assume that *tag i* is seen only in subset *S_i_* ⊆ *W_i_* of all epochs in the window *Wi.* Assuming that, the tag probabilities within an approximately sized window calculated using Equation (1), are relatively homogeneous, taking their average will give a valid estimate of the actual probability of *tag i* during window *W_i_* [16]. Therefore, the average empirical read rate $p_{i}^{\text{avg}}$ over the observation epochs is given is given by Equation (2) [16]:

(2) $$p_{i}^{\text{avg}}=\left(1/\left|S_{i}\right|\right)\cdot \sum_{t\in S_{i}}p_{i,t}$$

Also *S_i_* can be seen as a binomial sample of epochs in *W_i_i.e.*, a Bernoulli trial with probability $p_{i}^{\text{avg}}$ for success and |*S_i_*| as a binomial random variable with binomial distribution $B\left(w_{i},p_{i}^{\text{avg}}\right)$. Hence, from standard probability theory the expected value and variance of |*S_i_*| is given as:

$$E\left[\left|S_{i}\right|\right]=w_{i}\cdot p_{i}^{\text{avg}}\text{and Var}\left[\left|S_{i}\right|\right]=w_{i}\cdot p_{i}^{\text{avg}}\cdot \left(1-p_{i}^{\text{avg}}\right)\;\text{respectively .}$$

The derived binomial sampling model is then used to set the window size to ensure that there are enough epochs in the window *W_i_* such that *tag i* is read if it does exist in the reader's range. Setting the number of epochs within the smoothing window according to Equation (3) ensures that *tag i* is observed within the window *W_i_* with probability >1 − *δ* [16]:

(3) $$w_{i}\geq \left\lceil \left(1/p_{i}^{\text{avg}}\right)\text{ln}\left(1/\delta \right)\right\rceil$$

#### 3.1.2. Adaptive Window Size Adjustment

In order to balance between guaranteeing completeness and capturing tag dynamics the WSTD algorithm uses simple rules, together with statistical analysis of the underlying data stream, to adaptively adjust the cleaning window size.

**Figure 1.**
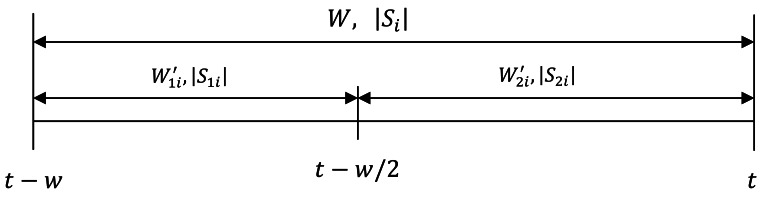
Illustration of the sub-ranges in the smoothing window.

Assume *Wi* = (*t* − *w_i_, t*) is *tag i* current window, and let *W*_1_*_i_′* = (*t* − *w_i_, t* − *w_i_/*2) denote the first half of window *Wi* and *W*_2_*_i_′* = (*t* − *w_i_/*2, *t*) denote the second half of the window *Wi*. Let |S_1i_| and|S_2i_| denote the binomial sample size during *W*_1_*_i_′* and *W*_2_*_i_′* respectively. Note that the mid epoch (*i.e.*, epoch at *t* − *w_i_/*2) in inclusive on both range as shown in Figure 1.

*Rule 1*: Similar to SMURF, variation within the window is detected if the number of observed readings is less than the expected number of readings (*i.e.*, $\left|S_{i}\right|<w_{i}\cdot p_{i}^{\text{avg}}$) and there is statistically significant variation in the tag observations using the Central Limit Theorem (CLT) $\left|\left|S_{i}\right|-w_{i}p_{i}^{\text{avg}}\right|>2\cdot \sqrt{w_{i}p_{i}^{\text{avg}}\left(1-p_{i}^{\text{avg}}\right)}$. However, we noted that this variation within the window could also be caused by missing tags and not necessarily only due to transition. Hence, to reduce the number of false positive due to transition and the number of false negative readings, which will be further introduced in case of wrong transition detection, the window size is reduced additively by reducing the window size by two epochs.

To improve the transition detection mechanism for the mobile tags we combine the mobile detection mechanism together with the observations of the second half of the window|*S*_2_*_i_*| to estimate when the tag is exiting the detection range. The slope of the best-fit line using the least squares fitting with the observed *tag i* probabilities in the window $\left(\frac{\Delta p_{i,t}}{\text{epochs}}\right)$ is used to determine if the tag is moving out. If the tag is detected with consistently falling *p_i,t_*, within the window it is inferred as the tag is moving out. Hence, the negative slope of the best-fit line indicates that the tag is moving out.

*Rule 2*: If the tag is moving out and it was not detected in the second half of the window (*i.e.*, |*S_2i_*| = 0) the tag is assumed to have exited or is exiting the detection range. In this case the window size is halved to reduce the false positive readings. One weakness of this rule is that premature exit transition detection will also lead to a false negative reading due to a small window size.

*Rule 3*: The window size is increased if the computed window size using Equation (3) is greater than the current window size and the expected number of observation samples is less than the actual number of observed samples (*i.e.*, $\left|S_{i}\right|>w_{i}p_{i}^{\text{avg}}$). Low expected observation samples indicates that the probability of detection $p_{i}^{\text{avg}}$ is low, in this case we need to grow the window size to give more opportunity for the poor performing tag to be detected. Otherwise, if the expected observation sample is equal or greater than the actual sample size it means that, the $p_{i}^{\text{avg}}$ is good enough and we do not have to increase the window size. This rule ensures that the window size is increased only when the read rate is poor.

**Figure 2.**
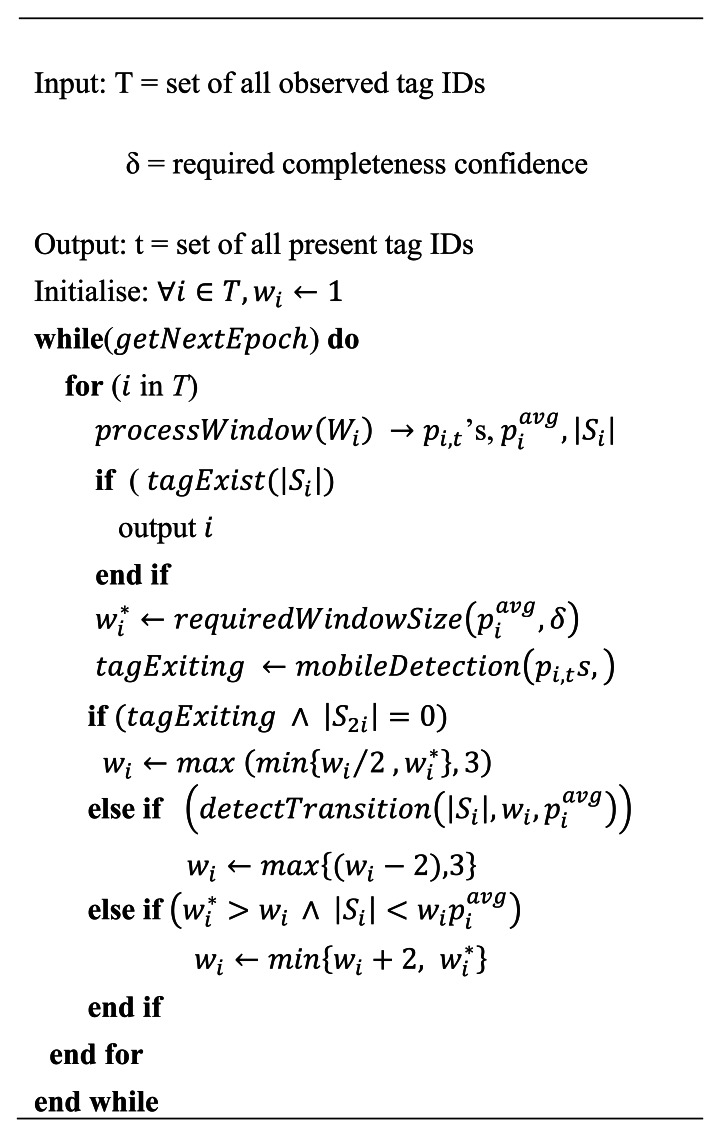
WSTD individual tag cleaning algorithm.

Figure 2 shows a pseudo-code description of the WSTD adaptive per tag cleaning algorithm. Each individual tag is cleaned in its own window. The rules described above are used to adjust the tag's cleaning window size adaptively based on the statistical analysis of the underlying tag observations. Initially all new detected tag's windows are set to one epoch, the window sizes are then adjusted according to their detection rates with minimum window size set to three epochs.

Setting the minimum window size to three epochs strikes a balance between maintaining the smoothing effect of the algorithm and reducing the false positive errors. Similar to SMURF, WSTD also slides its window per single epoch (read cycle) and produces output readings corresponding to the midpoint of the window after the entire window has been read.

### 3.2. Multi-Tag Aggregate Cleaning

Some applications do not require information for each individual tags, but only need to track the number of tags in the detection region. These types of applications typically track large populations of tags. For instance, a retail store monitoring application may only need to know when the count of items on the shelf or store drop below a certain threshold level.

The per tag cleaning method could be used to clean tags in such scenarios, where by each tag in the population is individually cleaned and their result is aggregated across individual smoothing filters for each epoch. However, this solution can be highly affected by poor performing tags especially in the static environment. The per tag cleaning algorithm adapts the window size for each individual tag and because window sizes for individual tags might be different, based on their detection rates, the decision on whether the tag is present or not is taken at different epochs. Therefore, due to different window sizes, the tags that are not ready for processing (*i.e.*, the readings for all epochs in its window have not be accumulated) will delay the output. To avoid this limitation caused by low performing tags, the multi-tag cleaning algorithm uses the same smoothing window for all the tags together with a statistical estimation technique to accurately estimate the tags population count without cleaning on a per-tag basis.

As with individual tag observation, the smoothing window size plays a critical role in capturing the underlying tag's population aggregate. A large window ensures that the tags are observed and aggregated with high probability, but a small window is also desired to ensure that variability in the population count is adequately captured.

The multi-tag cleaning mechanism uses some of the concepts proposed in SMURF whereby the Horvitz-Thompson (HT) estimator [24] also known as the π-estimator together with unequal-probability random sampling model is used to approximate the population aggregates. As with the per-tag cleaning method, the multi-tag cleaning mechanism also views each epoch as an independent Bernoulli trial with probability $p_{i}^{\text{avg}}$ for success, where $p_{i}^{\text{avg}}$ denotes the average empirical sampling probability for *tag i* during window *_W_* derived from the reader's tag list information using Equation (2).

Let *Sw* denote the sample of distinct tags read over the current smoothing window and let $p^{\text{avg}}=\left(1/\left|S_{W}\right|\right)\cdot \sum_{i\in S_{W}}p_{i}^{\text{avg}}$ denote the average per-epoch sampling probability over all observed tags. Following the similar rationale used in the per-tag cleaning, to ensure that the underlying tag population is read with high probability (≥1 − *δ*) we set the upper bound of the smoothing window size for multi-tag aggregate at *w* = [(1/*p^avg^*)ln(1/*δ*)]. According to binomial distribution, the probability of reading *tag i* at least once during window *w* = |*W*| is estimated as one minus probability of not detecting *tag i* in all the trials $\pi_{i}=1-{\left(1-p_{i}^{\text{avg}}\right)}^{w}$. Let *S_W_* ⊆ {1, …, *N_W_*} denote the subset of distinct observed (*i.e.*, sampled) RFID tags over the window *W* and *Nw* denote the true tags count. The π-estimator for the population count based on the sample *Sw* is defined as ${\hat{N}}_{W}=\sum_{i\in S_{W}}\frac{1}{\pi_{1}}$. The π-estimator uses the sampling probability π*_i_* to weigh the responses in estimating the population total. The poor performing tags with lower response probability are given higher weights while higher probability responses are given lower weights. The π-estimator gives unbiased estimation of tag population *N̂_W_* with its estimated mean and variance given as E(*N̂_W_*) = *N_W_* and $\hat{V}ar\left({\hat{N}}_{W}\right)=\sum_{i\in S_{W}}\frac{1-\pi_{i}}{\pi_{i}^{2}}$, respectively.

#### 3.2.1. Adaptive Window Size Adjustment

The WSTD cleaning algorithm employs the random-sampling model and π-estimator concepts proposed in SMURF together with comparison of the two-window sub-range estimated tag counts to dynamically adapt its smoothing window size. Transitions are detected as statistically significant changes in aggregate estimates over sub-ranges of its current smoothing window. The transition detection model used is the main difference between our multi-tag cleaning algorithm and the SMURF multi-tag cleaning algorithm.

Assume *W* = (*t* − *w, t*) is current window, and let *W*_1_*′* = (*t* − *w, t* − *w/2*) denote the first half of window *W* and *W*_2_*′* = (*t* − *w/2, t*) denote the second half of the window *W*. Let ${\hat{N}}_{W_{1}^{\prime }}$ and ${\hat{N}}_{W_{2}^{\prime }}$ denote the π-estimators for tag population counts during *W*_1_*′* and*W*_2_*′* respectively. Note that the mid epoch (*i.e.*, epoch at *t* − *w/2*) is inclusive in both ranges. The mid-point divides the window such that the numbers of epochs are equally spaced on either side of the window and this requires the use of an odd number window size. The transition is detected if there is significant change in tag counts between these two ranges.

In the SMURF multi-tag cleaning algorithm the transition is detected as a statistically significant transition in population count has occurred in the second half of the window compared to the whole window population count by using CLT condition $\left|{\hat{N}}_{W}-{\hat{N}}_{W_{2}^{\prime }}\right|>2\left(\sqrt{\text{Var}\left({\hat{N}}_{W}\right)}+\sqrt{\text{Var}\left({\hat{N}}_{W_{2}^{\prime }}\right)}\right)$. However in our model the transition is detected as a significant change in the population count by comparing the count estimates in the first half and the second half of the window by using the expression $\left|{\hat{N}}_{W_{1}^{\prime }}-{\hat{N}}_{W_{2}^{\prime }}\right|>2\left(\sqrt{\text{Var}\left({\hat{N}}_{W_{1}^{\prime }}\right)}+\sqrt{\text{Var}\left({\hat{N}}_{W_{2}^{\prime }}\right)}\right)$. Our experimental results verified that using the comparison of the sub-range population count estimates to detect population count variation within the window, gives a more accurate transition detection technique than comparison between full window count and the sub-range count estimates used by SMURF. SMURF detection condition detects any significant variation within the window, however, for transition detection mechanism we are more interested in detecting the edge transition, which signals that the tag is either entering or leaving the detection range and respond accordingly.

By comparing the population count of the two window sub ranges, it is possible to determine when the tag is exiting and entering the detection range eliminating the need to use mobile detection algorithm as proposed by SMURF. In the environment where tags are mobile there are two scenarios: one is tags exiting the detection range and the second is tags entering the detection range.

We use simple rules to detect when these transitions occur by comparing the estimated tag count in the two window sub ranges. The tags are said to be exiting the detection range if the transition is detected and there is more estimated tag counts in the first half of the window than in the second half of the window (*i.e.*, ${\hat{N}}_{W_{1}^{\prime }}>{\hat{N}}_{W_{2}^{\prime }}$). In this case, the window size is reduced multiplicatively (*i.e.*, divided in half) to circumvent false positive readings. Similarly the tag is said to be entering the detection region if transition is detected and there is more estimated tag count in the second half of the window than in the first half of the window (*i.e.*, ${\hat{N}}_{W_{2}^{\prime }}>{\hat{N}}_{W_{1}^{\prime }}$). In this case, if the required window size is greater than twice the current window size, the window size is increased multiplicatively (*i.e.*, doubled) if not, the window size is additively increased by two epochs. This is because as tag enters the detection range it is assumed to be on the far end of reader's detection ranges *i.e.*, long distance from the reader's antenna. Increasing the window size gives more opportunity even for the weak performing tags to de detected.

**Figure 3.**
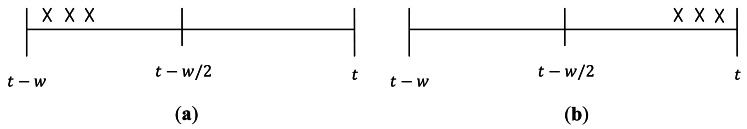
Illustration of mobile tag window sub-range as tags enters and exits the detection range. (**a**) Tags exiting; (**b**) Tags entering.

When the tags are leaving and entering the detection range, false positive readings will be produced regardless of the window size because the readings are interpolated throughout the window. This problem is more prone to the bigger windows. To reduce false positive readings under these scenarios we made two estimation assumptions. These approximation assumptions are used to detect when the tag(s) completely exit(s) the detection range and when the tag(s) just entered the detection region as illustrated in Figure 3. In the first assumption, the tag(s) are said to have exited the reader's detection range if the overall window tag count is not zero, but the second half of the tag population count is zero (*i.e.*, ${\hat{N}}_{W}>0\wedge {\hat{N}}_{W_{2}^{\prime }}=0$). This means that there was no tag observed in the second half of the window. In the second assumption, the tag(s) are said to have just entered the reader's detection range if the overall window tag count is not zero, but the first half of tag population count is zero (*i.e.*, ${\hat{N}}_{W}>0\wedge {\hat{N}}_{W_{1}^{\prime }}=0$). This means that there was no tag observed in the first half of the window (*t* − *w, t* − *w*/*2*).

Considering that the cleaning window size slides by the midpoint, we assume that the observed tags under these scenarios are more likely to be a false positive readings caused by a bigger window size. Therefore, tag(s) observed in these scenarios are dropped and the window size is reduced for an exiting scenario and increased appropriately for an entering scenario.

By taking advantage of the π-estimator, which scales-up the reading in the window to estimate the underlying tag population, we can reduce the window sizes to enhance transition detection, hence the minimum window size can be reduced to 1 epoch. We introduce another estimation condition, which we call *strong region detection*. The aim of *strong region detection* is to detect when the tags within the window are observed with high probability of detection and when there is no significant variation in tag population within the two window sub-ranges.

Let *S_i_* be a binomial sample of epochs in the current window *W* in which a single tag is observed and $p_{i}^{\text{avg}}$ be the average read rate as defined in the per-tag cleaning approach. Let *Sw* denote the sample of distinct tags read over the current smoothing window and $p^{\text{avg}}=\left(1/\left|S_{W}\right|\right)\cdot \sum_{i\in S_{W}}p_{i}^{\text{avg}}$ denote the average sampling probability over all observed tags and $S^{\text{avg}}=\left(1/\left|S_{W}\right|\right)\cdot \sum_{i\in S_{W}}S_{i}$ denote the average sample of epochs in the window in which the tags where observed.

The tags are then said to be observed in the strong detection region if the following condition holds $\left(p^{\text{avg}}>\left(1/W\right)\cdot S^{\text{avg}}\right)\wedge \left(\left|{\hat{N}}_{W_{1}^{\prime }}-{\hat{N}}_{W_{2}^{\prime }}\right|<\left\lceil 0.05\cdot \text{min}\left({\hat{N}}_{W_{1}^{\prime }},{\hat{N}}_{W_{2}^{\prime }}\right)\right\rceil \right)$. The second portion of the logical condition tests if the two window sub-range estimates have a relatively small difference of less than 5% of the lowest estimated tag counts. If the condition holds the window size is reduced by two epochs.

**Figure 4.**
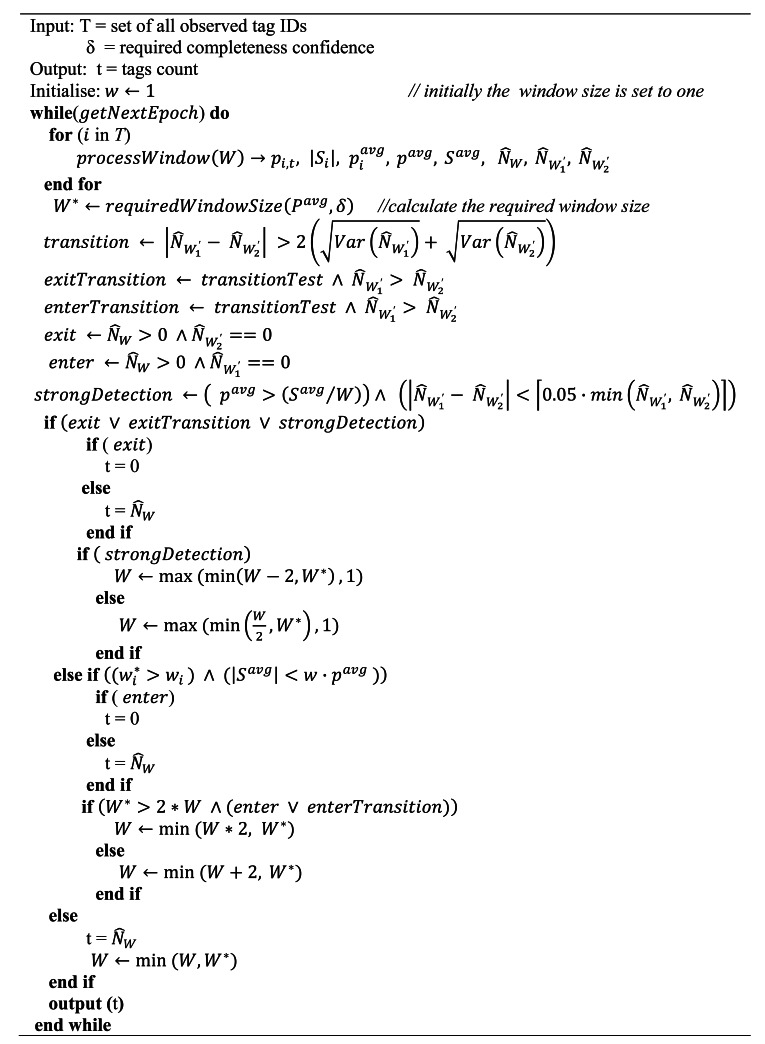
WSTD-π multi-tag cleaning algorithm.

Figure 4 shows a pseudo-code description of the adaptive multi-tag cleaning algorithm. All the tags are cleaned using the same window. Similar to per-tag cleaning, the smoothing-window size is systematically adjusted based on the analysis of the observed tags binomial-sampling data and the transition is detected by comparing the window sub-range estimated population counts.

## 4. Experimental Evaluation

In this section we present our experimental evaluation of the proposed WSTD cleaning algorithms. The data sets for our experiments were generated by a synthetic data generator that simulates the operation of RFID readers under a wide variety of conditions using MATLAB. The generator is composed of two components. The first component simulates the movement of tags and the second component simulates tag detection by an RFID reader.

We investigated three tag movement behaviours. The first behaviour is that of static tag(s). This is simulated by randomly placing tags uniformly within the reader's detection region. This simulates static tagged items which are constantly monitored by the RFID system (*i.e.*, both tags and readers are static). The second behaviour is that of tags moving with the same velocity. This simulates grouped tags, such as tagged items on a trolley or conveyor belt. The third behaviour is that of tags moving with different velocity. This behaviour simulates tracking environments, such as a digital work place where each tag displays independent random behaviour. Tag movements are simulated by moving the tags in and out of the reader detection range between 0 and 6 m at varying speed. We set the maximum detection range to be 4.6 m (∼15 feet) between 4.6 and 6 m the tag is out of the detection range. The tag velocities are varied between 0 and 90 cm/epoch.

**Figure 5.**
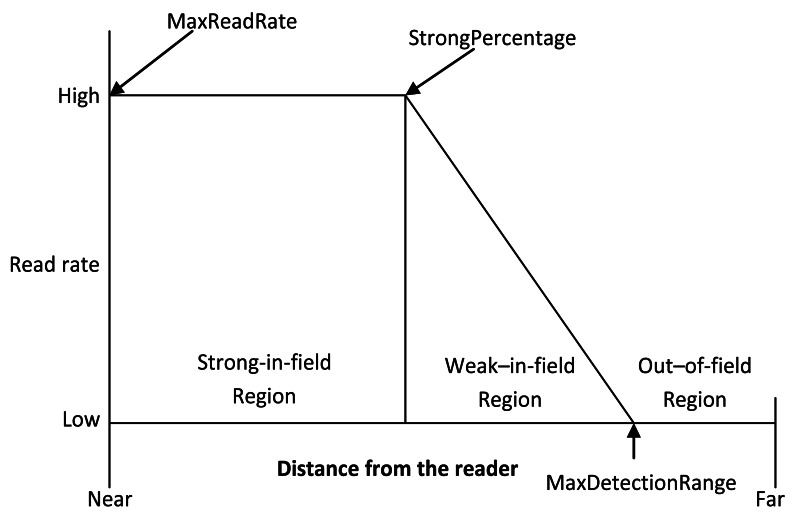
Passive RFID tag-reader detection regions.

The reader detection model is based on the RFID tag-reader detections regions. Generally there are three distinct regions of operations of a passive RFID reader tag system: strong-in-field, weak-in-field and out-of-field regions [5,16,25], as illustrated in Figure 5.

In the strong-in-field region, the tag responds to most of the attempts from the reader. Thus the response rate in the strong-in-field region varies between 100% and 77% [25]. The tag performance then degrades gradually with increasing distance in the weak-in-field region. In the out-of-field region, the response rate goes down to 0%. The main difference on this detection pattern when the tags are operated in different environments lies in the percentage of the reader's detection range corresponding to its strong-in-field region. When the tags are operating in a controlled environment with low RF interference, the strong-in-field region corresponds to roughly 75% of the full detection region, where as it makes only 25% of the range in the noisy environment with high RF interference [16]. Based on these observations, we derive a simplified reader detection model shown in Equation (4):

(4) $$p_{i,t}(x)=\{\begin{array}{ccc} \text{MaxReadRate}, & & x<\text{StrongPercentage}\\ \frac{\text{MaxReadRate}(x-\text{MaxDetectionRange})}{\text{StrongPercentage}-\text{MaxDetectionRange}}, & \text{StrongPercentage} & \leq x\leq \text{MaxDetectionRange}\\ 0, & & x>\text{MaxDetectionRange} \end{array}$$

In our experiments we used a maximum read rate (*MaxReadRate*) of 95% which is a read rate within the strong-in-field region. Maximum detection range (*MaxDetectionRange*) of 4.6 m and varied the strong-in-field percentage (*StrongPercentage*) and the distance between the tag and the reader (*x*). Varying the *StrongPercentage* parameter simulates the factors that affect the tag detection rates such as tag orientation and the RF interference while varying the distance (*x*) parameter simulates the tag-reader signal attenuation with distance. In the per-tag cleaning algorithms we used 25 tags while in the multi-tag aggregate algorithms we used 100 tags and the data were generated for 2,000 read cycles (epochs). We compare the performance effectiveness of the WSTD algorithms with that of SMURF using the generated synthetic data sets.

### 4.1. Individual Tag Cleaning

#### 4.1.1. Experiment 1: Environment Reliability with Randomly Moving Tags

**Figure 6.**
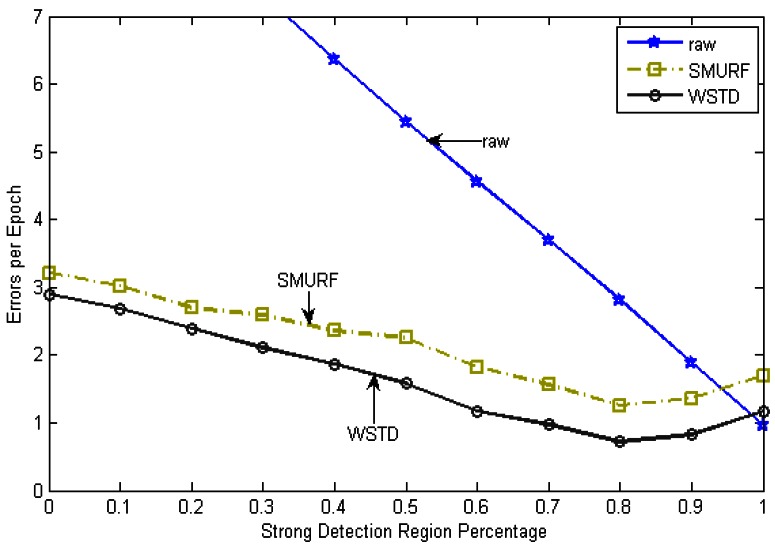
Average errors per epoch as strong-in-field region percentage is varied.

In this experiment we determine how each technique reacts to different levels of environment unreliability with the randomly moving tags. Each tag is moved with its own random velocity between 0 to 90 cm/epoch and after every 100 epochs on average the tag change its state from moving to rest state and vice versa. When the tag resumes movement it chooses another random velocity, this movement pattern is referred as *Fido* in [16]. The strong-in-field region percentage is varied between 0 and 100%. The lower *StrongPercentage* corresponds to unreliable environment and higher values of *StrongPercentage* corresponds to a more controlled environment. At each *StrongPercentage* we measure the average errors produced by each scheme. The average error per epoch is calculated as $\sum_{i=1}^{\text{NumEpochs}}\left({\text{FalsePositive}}_{i}+{\text{FalseNegative}}_{i}\right)/\text{NumEpochs}$. Figure 6 shows the result of this experiment, the “raw” trace is truncated to enable clear view of other traces.

**Figure 7.**
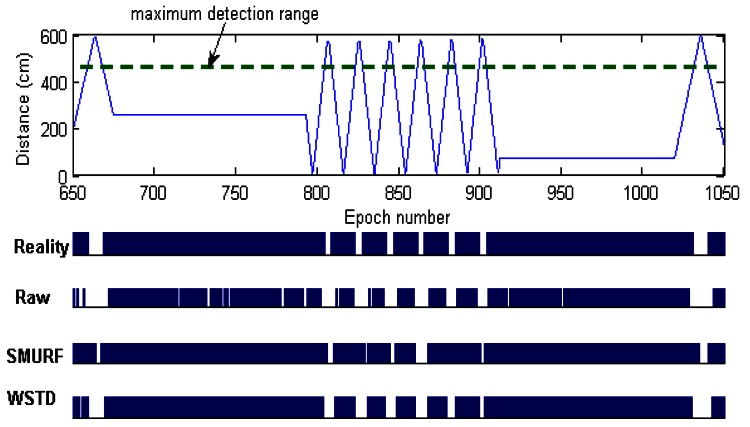
Comparison of WSTD and SMURF schemes' transition detection mechanisms as a tag moves at random velocity.

**Figure 8.**
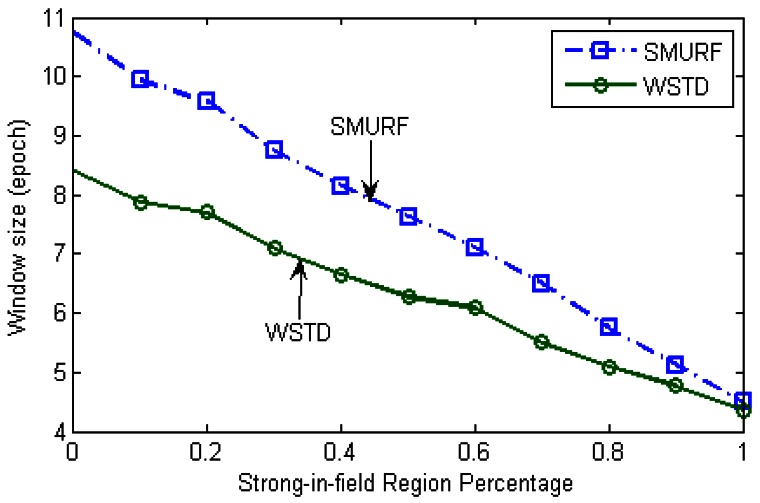
Comparison of WSTD and SMURF schemes' cleaning-window sizes as the environment noise is varied.

The WSTD scheme performs better than SMURF producing an improvement of approximately 25% less overall error in comparison to that produced by SMURF. This performance is attributed to its improved transition detection mechanism as shown in Figure 7. Comparing their cleaning-window sizes, WSTD uses a smaller window size in comparison to that used by SMURF as shown in Figure 8. The cleaning-window sizes decrease along with the decrease in environment noise.

**Figure 9.**
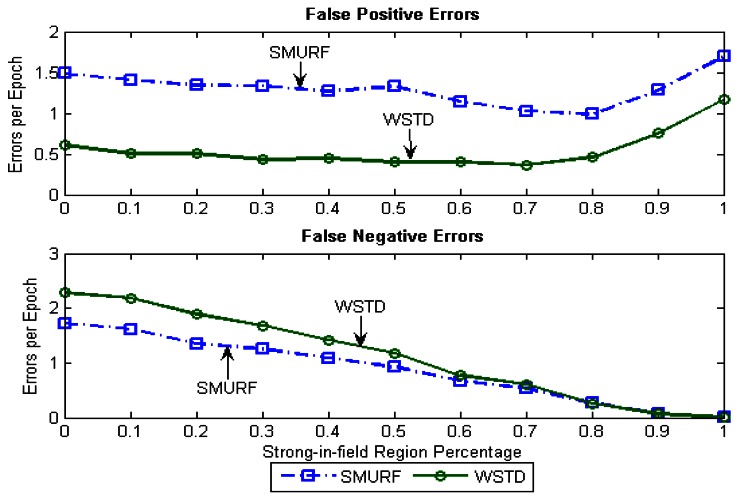
WSTD and SMURF schemes' false positive and false negative error contribution as the environment noise is varied.

Because of its small window size, WSTD is more efficient in detecting transition than SMURF; however, it also produces slightly more negative errors than SMURF as show in Figure 9. The increase in false negative errors in the noisy environment by WSTD can be associated with the premature transition detection by *rule2* of the WSTD algorithm. As the noise decreases, their performance in compensating for missed readings become competitive and their difference decreases.

#### 4.1.2. Experiment 2: Effect of Tag Speed

**Figure 10.**
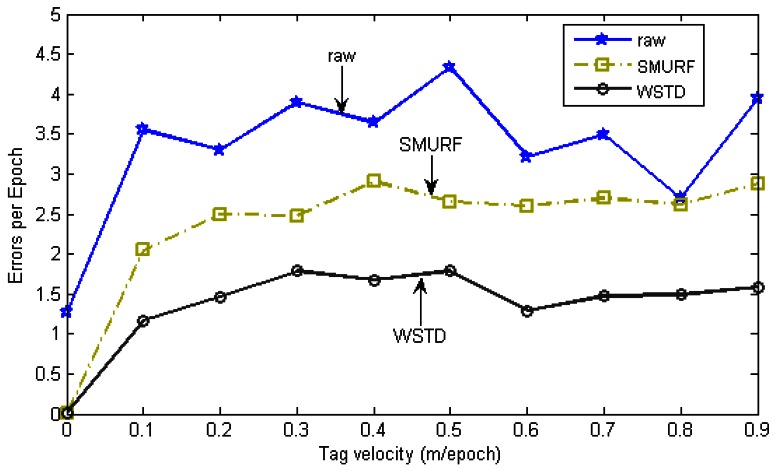
Average errors per epoch as tag velocity varies.

**Figure 11.**
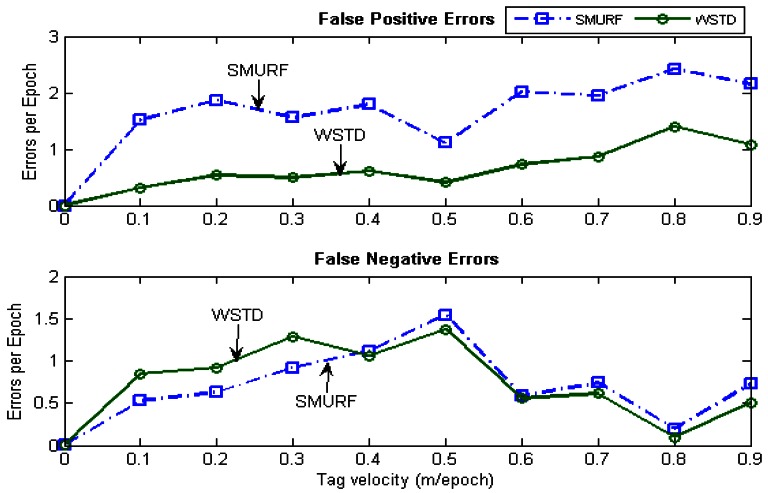
WSTD and SMURF schemes' false positive and false negative error contribution as the tag velocity is varied.

The effectiveness of the individual tag cleaning schemes are then compared as the tag velocity is varied. The *StrongPercentage* parameter is fixed at 70% to represent the controlled environment and the tags are moved in and out of the detection range at the same constant velocity. The velocity is varied from 0 to 90 cm/epoch the average errors produced by each scheme were measured. Figure 10 shows the result of this experiment and Figure 11 shows their positive and negative error contributions as the tag velocities are varied.

**Figure 12.**
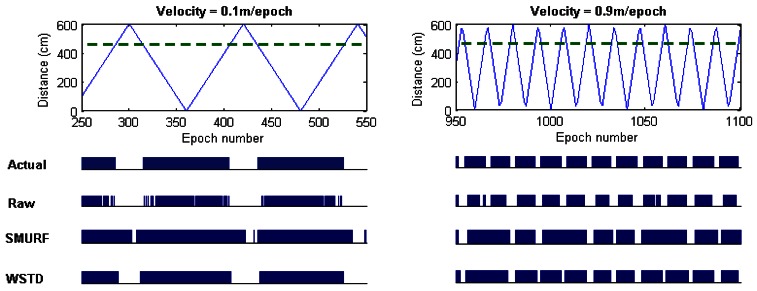
Comparison of WSTD and SMURF schemes' transition detection mechanisms as a tag moves at constant velocity.

**Figure 13.**
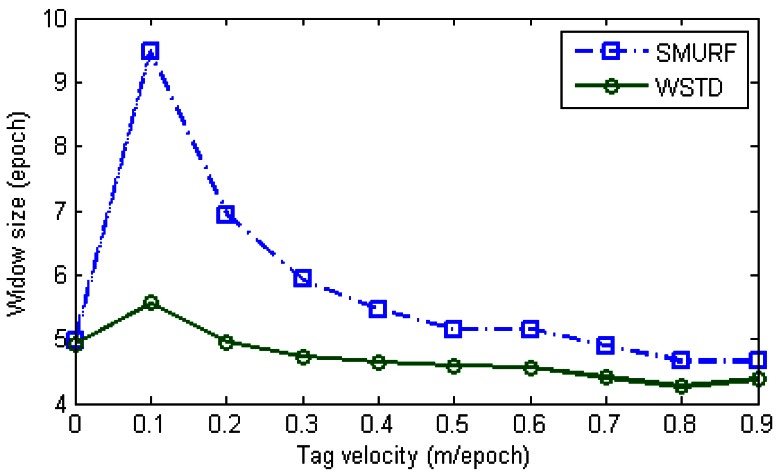
Comparison of WSTD and SMURF schemes' cleaning window sizes as the velocity is varied.

The WSTD scheme performs better than SMURF producing an improvement of approximately 30% less overall errors in comparison to that produced by SMURF. This performance improvement is attributed to its improved transition detection as shown in Figure 12, due to its use of small window sizes as shown in Figure 13. The cleaning-window sizes decreases with the increase in tag speed.

#### 4.1.3. Experiment 3: Environment Reliability with Static Tags

**Figure 14.**
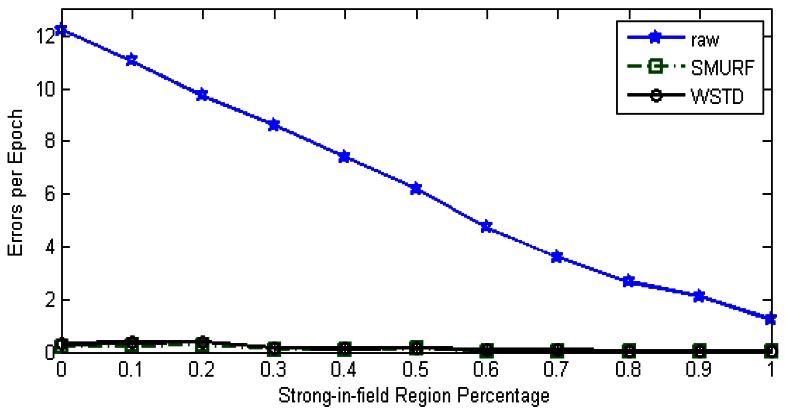
Average errors per epoch as strong-in-field region percentage is varied in the static tag environment.

**Figure 15.**
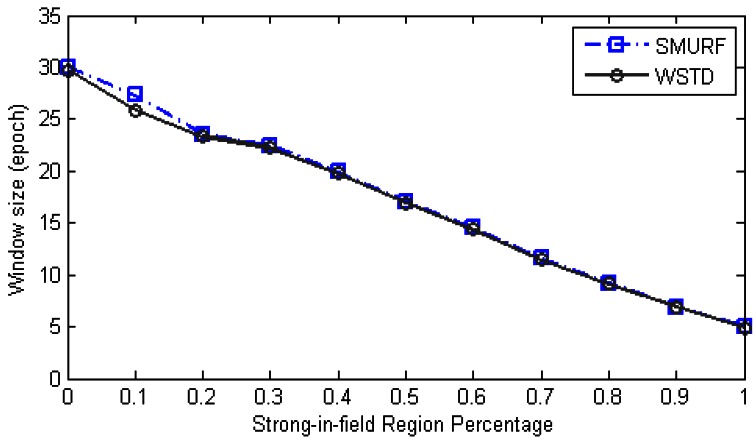
Comparison of WSTD and SMURF schemes' cleaning window sizes as the environment noise is varied.

We also evaluated the performance of different cleaning schemes in the environment where tags are stationary. To simulate this scenario we randomly distribute the tags uniformly within the detection range and varied the *StrongPercentage* parameter and measured the average errors produced by each scheme. Figure 14 shows the result of this experiment and Figure 15 shows the average cleaning-window sizes for the SMURF and WSTD schemes as the environment noise is varied.

In the static environment, both WSTD and SMURF schemes exhibit closely matching performances which can be attributed to their use of similar cleaning-window sizes as shown in Figure 15. From this observation we can conclude that the main difference between WSTD and SMURF is in the transition detection mechanism, and WSTD performs better than SMURF in the mobile environment.

### 4.2. Tags Aggregate Cleaning

We then examined the cleaning techniques, which report the number of tags in the detection region instead of individual tag Id. The performances of different multi-tag aggregate cleaning schemes are compared as the tag movement and reliability of the environment are varied. Individual tag cleaning schemes, SMURF and WSTD, reports the number of distinct tags in each epoch, while WSTD-π scheme uses the π-estimator to estimate the number of distinct tags within the window.

The evaluation metric used for multi-tag cleaning is the root mean square (RMS) error of the count of reported tags compared to the actual tag count. The RMS error is calculated using Equation (5):

(5) $$\text{RMS Error}=\sqrt{\frac{\sum_{i=1}^{\text{NumEpochs}}{\left({\text{ReportedCount}}_{i}-{\text{ActualCount}}_{i}\right)}^{2}}{\text{NumEpochs}}}$$

We also compared the mean error of the estimated tag count to see the contribution of the overestimate and underestimate tag count using Equation (6) and (7), respectively:

(6) $$\text{OverEstimateErrors}=\frac{\sum_{i=1}^{\text{NumEpochs}}{\text{ReportedCount}}_{i}-{\text{ActualCount}}_{i}}{\text{NumEpochs}}$$

(7) $$\text{UnderEstimateErrors}=\frac{\sum_{i=1}^{\text{NumEpochs}}{\text{ActualCount}}_{i}-{\text{ReportedCount}}_{i}}{\text{NumEpochs}}$$

#### 4.2.1. Experiment 4: Effect of Tag Speed on Tags Aggregate Cleaning

**Figure 16.**
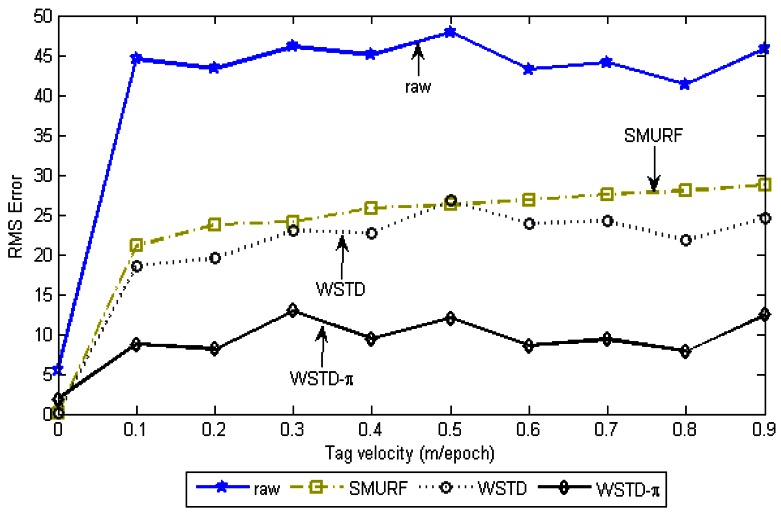
The RMS error of different cleaning schemes counting 100 tags as their velocities varies from 0 to 0.9 m/epoch in the noisy environment with the *StrongPercentage* parameter set to 25%.

We evaluate the tag count accuracy of the cleaning schemes as the tags' velocity is varied. The *StrongPercentage* parameter is fixed at 25% to represent the noisy environment and the tags are moved in and out of the detection range at the same velocity. The velocity is varied from 0 to 90 cm/epoch and we measured the RMS errors produced by each scheme. Figure 16 shows the result of this experiment.

**Figure 17.**
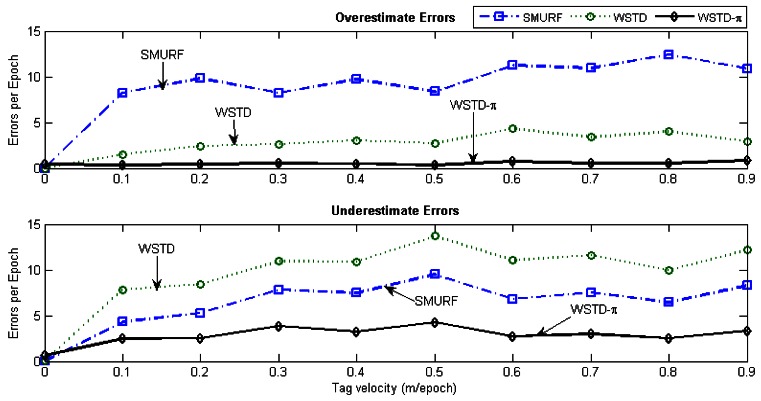
Variable window schemes overestimate and underestimate error contributions as the tags' velocity is varied.

**Figure 18.**
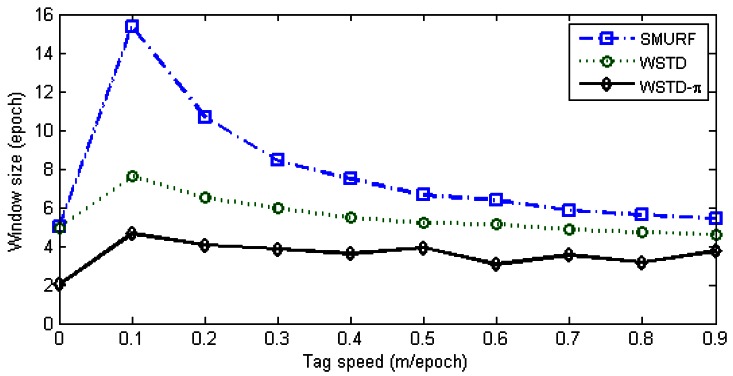
Comparison of variable window schemes' cleaning windows as the tags velocity is varied.

Figure 17 shows the average number of overestimate and underestimate tag counts for the cleaning schemes and Figure 18 shows their average cleaning-window sizes as the tags' velocity is varied. The WSTD-π scheme has the smallest number of underestimate and overestimate errors and it also uses the smallest average cleaning-window sizes compared to other variable window schemes. The WSTD-π small overestimate errors are attributed to its smaller window size while its small underestimate errors are attributed to its use of π-estimator to estimate the number of tags.

**Figure 19.**
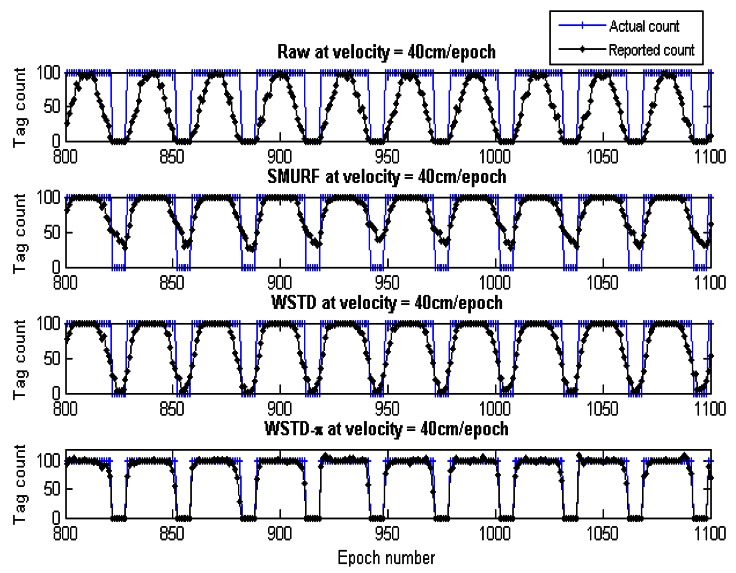
Comparison of variable window-cleaning schemes' reported tags count with the actual tag count. All the tags move with the same velocity of 0.4 m/epoch.

Although we used an average number of overestimate and underestimate tag counts per epoch as a metric to compare the performance of these schemes, we noticed that most of the undercounting errors occur during the transition periods. This is caused by the nature of algorithms whereby the window size is reduced when an exit transition is detected. While this measure limits the false positive errors, it also leads to false negative errors due to resulting small window size in case of premature exit transition detection. In addition, when the tags are entering the detection region on the far edge of the detection range due to a small window size and a weak read rate, leads to a high number of undercounting errors. Figure 19 shows the comparison of the reported estimated tags count and that of the actual tag count for the three variable window schemes SMURF, WSTD and WSTD-π with the tags moving at a velocity of 0.4 m/epoch. WSTD-π provides close accurate tag-count estimation compared to other schemes.

#### 4.2.2. Experiment 5: Effect of Environment Reliability with Randomly Moving Tags

**Figure 20.**
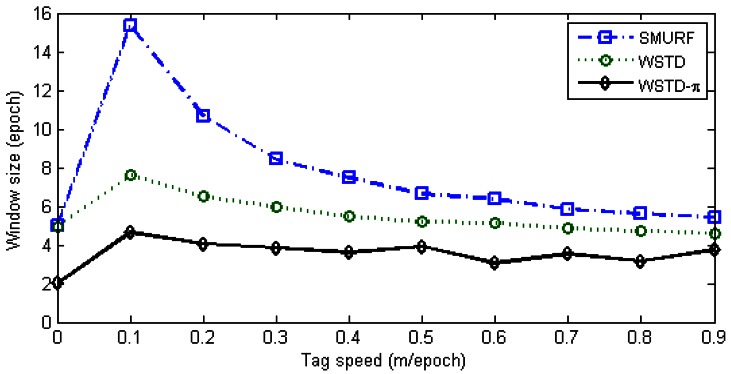
RMS errors of the tags count for different cleaning schemes as the *StrongPercentage* parameter is varied with each tag moving with its own velocity.

In this experiment we determine how each multi-tag cleaning technique reacts to different levels of the environment's unreliability with the randomly moving tags. The experimental parameters are the same as the ones used for the individual tag-cleaning experiment, except that in this experiment we used 100 moving tags instead of 25 tags. The strong-in-field region percentage is varied between 0 and 100% and at each *StrongPercentage* we measure the RMS errors produced by each scheme. Figure 20 shows the result of this experiment, “raw” trace is truncate to enable a clear view of other traces.

**Figure 21.**
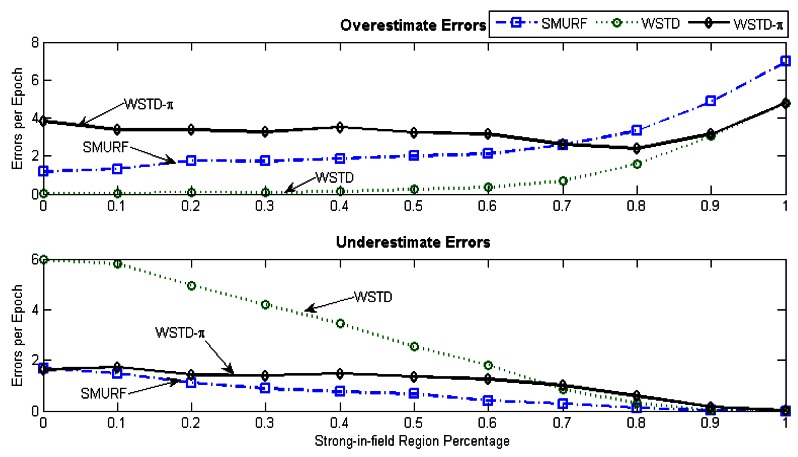
Variable window schemes' average overestimate and underestimate error contributions as the *StrongPercentage* parameter is varied with each tag moving with its own velocity.

**Figure 22.**
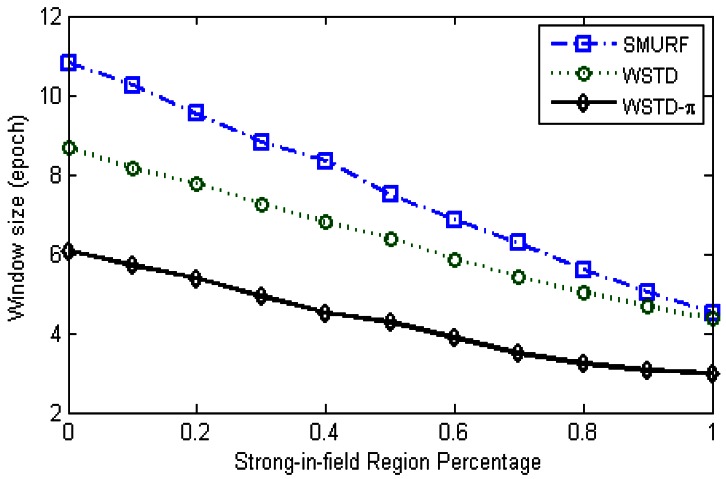
Comparison of variable window schemes' cleaning window sizes as the *StrongPercentage* parameter is varied.

Figure 21 shows average number of overestimate and underestimate tag counts and Figure 22 shows the average cleaning-window size of the variable window schemes as the environment noise is varied.

**Figure 23.**
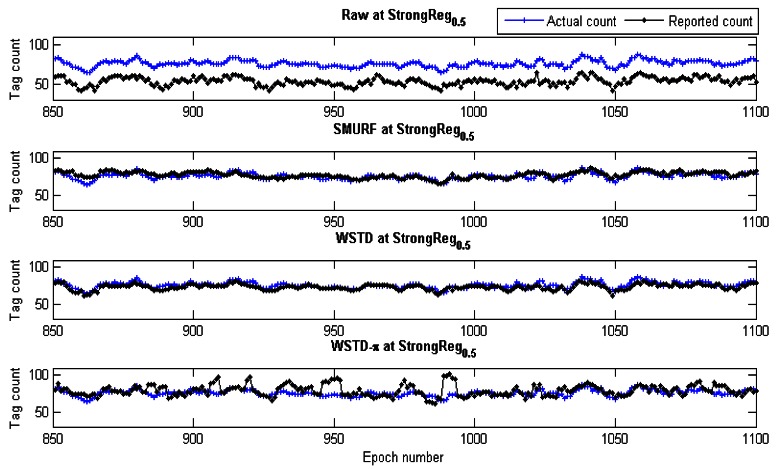
Comparison of variable window-cleaning schemes' tags count with the actual tag count. Each tag moves with its own velocity.

From Figure 21 looking at per-tag cleaning schemes; SMURF has consistently more overestimate errors than WSTD, while in a noisy environment WSTD has more underestimate errors than SMURF. However, as the noise decreases and the reader produce more reliable data, the WSTD-undercount errors also decrease and at a highly controlled environment, its performance outperforms that of SMURF. WSTD-π on the other hand, consistently produces relatively stable overestimate errors irrespective of the environment condition; however, its underestimate errors decrease with the decrease of the environment noise (see Figure 21). These constant overestimate errors might be caused by the fact that WSTD-π uses the same window size to clean all the tags. In this scenario each tag moves randomly in and out of detection range with random velocities. WSTD-π transition mechanism is not able to effectively detect transition in this scenario; as a result the π-estimator overestimates the tags' count. The per-tag variable window cleaning schemes—SMURF and WSTD—outperform a single variable window scheme—WSTD-π—in the noisy environment because they clean and adjust their window size for each single tag independently depending on its individual tag behaviour and how the environment is affecting that particular tag. Figure 23 shows the comparison of the reported estimated tags' count and that of the actual tag count for these three variable window schemes. The tags are operating in the semi-controlled environment with *StrongPercentage* parameter set to 50%. Hence, this experiment results demonstrate that in the environment where each tag displays its own independent random behaviour the best result is obtained by adjusting each individual tag cleaning window independently.

#### 4.2.3. Experiment 6: Effect of Environment Reliability with Static Tags

**Figure 24.**
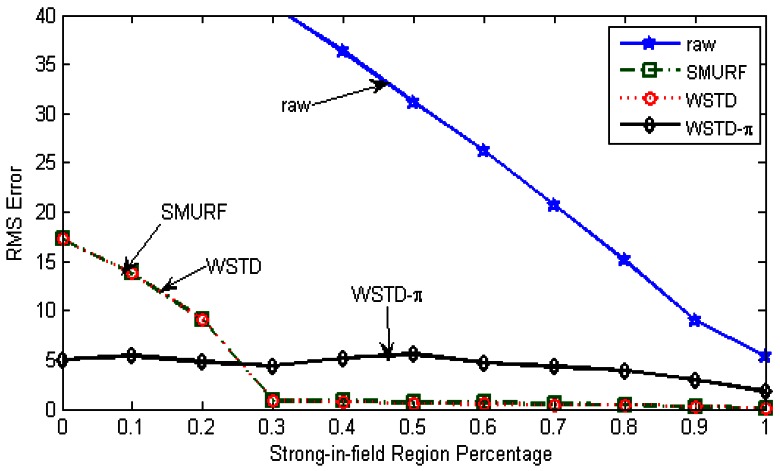
The RMS error of different cleaning schemes as the *StrongPercentage* parameter varies in the static tags' environment.

We also evaluated the performance of different sliding-window based multi-tag cleaning schemes in the environment where tag(s) are stationary. To simulate this scenario we randomly distributed 100 tags uniformly within the detection range and varied the *StrongPercentage* parameter and measured the root mean square error produced by each scheme. Figure 24 shows the result of this experiment, the “raw” trace is truncate to enable clear view of other traces.

Similar to the per-tag cleaning in the static environment scenario, SMURF and WSTD schemes have the same performance in cleaning the tag aggregations in the static environment as shown in Figure 24.

**Figure 25.**
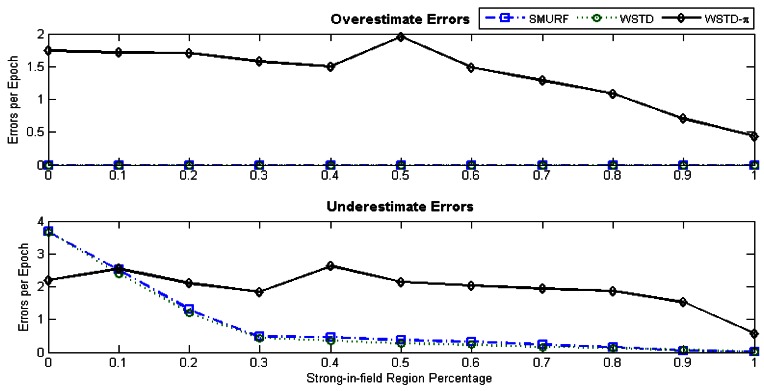
Variable window schemes' average overestimates and underestimates error contributions as the *StrongPercentage* parameter is varied in the static tag environment.

Since they both count the distinct tag available within each tag's cleaning window they have no overestimate errors and their underestimate decrease with the decrease in noise as shown in Figure 25. In a highly noisy environment the performance of per tag cleaning schemes is highly affected by the poor performing tags on the far edge of the detection region. In the noisy environment the far edge static tags cleaning window grows linearly with distance and becomes very large (results not shown). Because the cleaning window sizes for individual tags might be different based on their detection rates, the decision at whether the tag is present or not is taken at different epochs. Therefore, the tags are not ready for processing (*i.e.*, the readings for all epochs in its window have not been accumulated) will delay the output and highly affect the performance of the scheme.

**Figure 26.**
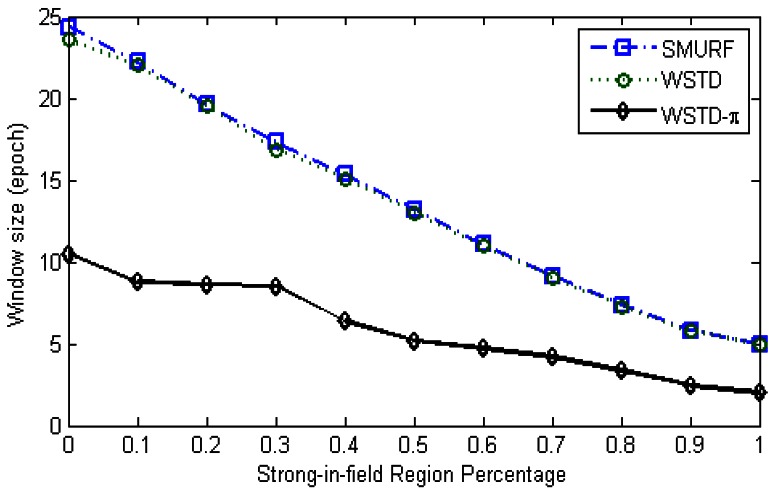
Comparison of variable window schemes' cleaning-window sizes as the *StrongPercentage* parameter is varied in the static tag environment.

On the other hand, the WSTD-π scheme, which estimates the tag count based on tag detections and π-estimator, produces both underestimate and overestimate errors as shown in Figure 25. Its performance increases with the reduction of environment noise with both its overestimate and underestimate errors decreasing. The WSTD-π scheme uses the smallest cleaning-window sizes compared to other schemes as shown in Figure 26. We can conclude that WSTD-π performance strikes a balance between accuracy and processing speed, by providing a considerably good estimate in a much shorter time.

## 5. Conclusions and Future Works

In this paper, we have proposed an adaptive window-based cleaning scheme called WSTD. WSTD uses binomial sampling concepts to calculate the appropriate window size and π-estimator to estimate the number of tags, and then uses the comparison of the two window sub-range observations or estimated tag counts to detect when transitions occurs within the window.

Our experimental results show that, in the mobile environment under a variable environment noise level, the WSTD scheme performs better than SMURF; producing an improvement of approximately 30% less overall errors than those produced by SMURF. This performance improvement is attributed to its improved transition detection mechanism. The WSTD scheme uses smaller window sizes compared to SMURF which means that WSTD also requires a shorter processing time than SMURF.

The WSTD algorithms have some limitations and we plan to investigate these limitations as part of our future work. Firstly, the WSTD transition detection algorithms are not very efficient in an extremely noisy environment. This is because in a noisy environment, the significant variation of tag observations or tag counts in the two windows sub-range, or lack of readings in one of the window sub-range, may be caused by missed readings rather than tag transition. Secondly, in the per-tag cleaning algorithm if the tag is detected with consistently falling probabilities within the window, it is inferred that the tag is moving out. However, if the tag is not completely moving out of detection range, and instead oscillate within the detection range, this mobile detection rule combined with observations of the second half of the window, in the noisy environment, may lead to false transition detection.

## References

[1] Finkenzeller K. (2003). RFID Handbook: Fundamentals and Applications in Contactless Smart Cards and Identification.

[2] Ahsan K., Shah H., Kingston P. (2010). RFID applications: An introductory and exploratory study. Int. J. Comput. Sci. Issues.

[3] Mitrokotsa A., Douligeris C., Zhang Y., Yang L.T., Chen J. (2010). Integrated RFID and Sensor Networks: Architectures and Applications. RFID and Sensor Networks: Architectures, Protocols, Security and Integrations.

[4] (2005). EPC Radio Frequency Identification Protocols Class-1 Generation-2 UHF RFID Protocol for Communications at 860 MHz–960 MHz; Standard Specification Version 1.2.0.

[5] Bolic M., Athalye A., Hao Li T., Bolic M., Simplot-Ryl D., Stojmenovic I. (2010). Performance of Passive UHF RFID Systems in Practice. RFID Systems: Research Trends and Challenges.

[6] Buettner M., Wetherall D. An Empirical Study of UHF RFID Performance.

[7] Aroor S., Deavours D. (2007). Evaluation of the state of passive UHF RFID: An experimental approach. IEEE Syst. J..

[8] Kawakita Y., Mitsugi J. Anti-Collision Performance of Gen2 Air Protocol in Random Error Communication Link.

[9] Darcy P., Stantic B., Sattar A. A Fusion of Data Analysis and Non-Monotonic Reasoning to Restore Missed RFID Readings.

[10] Trotter M.S., Durgin G.D. Survey of Range Improvement of Commercial RFID Tags with Power Optimized Waveforms.

[11] Rahmati A., Zhong L., Hiltunen M., Jana R. Reliability Techniques for RFID-Based Object Tracking Applications.

[12] Chen H., Ku W., Wang H., Sun M. Leveraging Spatio-Temporal Redundancy for RFID Data Cleansing.

[13] Mahdin H., Abawajy J. (2011). An approach for removing redundant data from RFID data streams. Sensors.

[14] Bashir A.K., Lim S.-J., Hussain C.S., Park M.-S. (2011). Energy efficient in-network RFID data filtering scheme in wireless sensor networks. Sensors.

[15] Shen H., Zhang Y. (2008). Improved approximate detection of duplicates for data streams over sliding windows. J. Comput. Sci. Technol..

[16] Jeffery S.R., Garofalakis M., Franklin M.J. Adaptive Cleaning for RFID Data Streams.

[17] Gonzalez H., Han J., Shen X. Cost-Conscious Cleaning of Massive RFID Data Sets.

[18] Song B., Qin P., Wang H., Xuan W., Yu G. bSpace: A Data Cleaning Approach for RFID Data Streams Based on Virtual Spatial Granularity.

[19] Rao J., Doraiswamy S., Thakkar H., Colby L.S. A Deferred Cleansing Method for RFID Data Analytics.

[20] Bornhoevd C., Lin T., Haller S., Schaper J. Integrating Automatic Data Acquisition with Business Processes—Experiences with SAP's Auto-ID Infrastructure.

[21] Gupta A., Srivastava M. (2004). Developing Auto-ID Solutions Using Sun Java System RFID Software.

[22] Massawe L.V., Aghdasi Kinyua J. The Development of a Multi-Agent Based Middleware for RFID Asset Management System Using the PASSI Methodology.

[23] Massawe L.V., Aghdasi F., Kinyua J. An Implementation of a Multi-Agent Based RFID Middleware for Asset Management System Using the JADE Platform.

[24] Lohr S.L. (1999). Sampling: Design and Analysis.

[25] Deavours D.D. (2004). A Performance Analysis of Commercially Available UHF RFID Tags Based on EPCglobal's Class 0 and Class 1; Specification Report 1.

